# Fosfomycin as an adjunctive agent against carbapenem-resistant *Acinetobacter baumannii*: an updated narrative review

**DOI:** 10.1007/s15010-026-02895-8

**Published:** 2026-07-27

**Authors:** Luca Pipitò, Eleonora Bono, Chiara Vincenza Mazzola, Antonio Cascio

**Affiliations:** 1 Department of Health Promotion, Mother and Child Care, Internal Medicine and Medical Specialties G D’Alessandro, University of Palermo, Palermo, 90133 Italy; 2 Infectious and Tropical Disease Unit, AOU Policlinico P. Giaccone, Palermo, 90127 Italy

**Keywords:** Carbapenem-resistant *Acinetobacter baumannii*, Fosfomycin, Combination therapy, Antimicrobial resistance, Antibiotic synergy, Clinical outcomes

## Abstract

Carbapenem-resistant *Acinetobacter baumannii* (CRAB) represents a critical therapeutic challenge due to limited effective treatment options and high associated mortality. Fosfomycin has been increasingly investigated as an adjunctive agent in combination regimens, despite its intrinsically reduced activity against *A. baumannii*. This narrative review summarizes current in vitro, in vivo, and clinical evidence on fosfomycin-containing therapies for CRAB infections. A total of 48 studies were included, encompassing in vitro (*n* = 27), animal (*n* = 2), and clinical investigations (*n* = 20). In one case, both in vitro and clinical data were reported. In vitro data suggest that fosfomycin may exhibit synergistic or additive activity when combined with several anti-CRAB agents, most consistently with sulbactam, imipenem, and colistin, although results are highly heterogeneous and method-dependent. Although limited, in vivo studies indicate improved bacterial clearance with fosfomycin-based combinations, particularly in pneumonia models. Clinical evidence remains largely observational and non-randomized, frequently involving critically ill patients receiving multiple combination therapies. While some multicenter studies, particularly from Italian cohorts, report improved clinical outcomes and survival associated with fosfomycin-containing regimens, especially in combination with cefiderocol, other studies do not confirm a clear benefit. Clinical trials have mainly demonstrated improvements in microbiological response and early infection control, without consistent effects on mortality. Overall, current evidence suggests that fosfomycin may provide additional benefit when incorporated into selected combination regimens for CRAB infections, although its independent contribution remains uncertain. Among the evaluated regimens, cefiderocol-fosfomycin combinations currently appear to be supported by the most encouraging observational clinical evidence, particularly regarding early clinical response and microbiological control.

## Introduction

Carbapenem-resistant *Acinetobacter baumannii* (CRAB) infections represent a major global health challenge for several reasons. First, *A. baumannii* has an extraordinary ability to acquire and develop antimicrobial resistance mechanisms, rendering many nosocomial isolates resistant to nearly all available antibiotics [1]. Second, CRAB is a leading cause of severe hospital-acquired infections and is associated with high in-hospital mortality [2, 3]. Third, therapeutic options for CRAB infections remain extremely limited, and no universally accepted standard of care currently exists [4].

Historically, polymyxins such as colistin have been widely used despite their well-known nephrotoxicity and neurotoxicity. More recently, newer agents such as cefiderocol and the combination sulbactam–durlobactam have been introduced; however, their use is constrained by limited global availability, emerging resistance, challenges in antimicrobial susceptibility testing, and a paucity of robust clinical data [4]. For these reasons, CRAB has been included among the ESKAPE pathogens by the Infectious Diseases Society of America (IDSA) and classified as a critical-priority pathogen by the World Health Organization (WHO) [5, 6].

Fosfomycin is a broad-spectrum bactericidal antibiotic that inhibits bacterial cell wall synthesis through irreversible inactivation of the MurA enzyme, acting at an early stage distinct from β-lactams. It demonstrates time-dependent killing with concentration-enhanced activity and a low degree of protein binding [7].

Intravenous fosfomycin is characterized by a small molecular weight and high hydrophilicity, allowing excellent tissue penetration, including into lungs, bone, and cerebrospinal fluid under inflamed conditions [7]. It is primarily eliminated unchanged via the kidneys, with a short half-life requiring repeated dosing or continuous infusion in severe infections [7]. Clinically, fosfomycin is generally well tolerated and has been increasingly used in combination regimens for the treatment of multidrug-resistant infections, where it may provide synergistic effects and help prevent the emergence of resistance [7, 8].

In antimicrobial combination studies, interactions between agents are commonly classified as synergistic, additive, indifferent, or antagonistic according to predefined pharmacodynamic criteria, most frequently based on the fractional inhibitory concentration index (FICI). Synergy generally refers to an antibacterial effect that exceeds the expected combined activity of the individual agents, indicating that the activity of one drug is potentiated by the presence of the other. By contrast, an additive effect describes an improvement in antibacterial activity that approximates the expected combined effect without evidence of potentiation beyond simple summation. Indifference indicates that the combination does not result in a meaningful enhancement or reduction of antibacterial activity compared with the most active individual agent, suggesting an absence of relevant pharmacodynamic interaction. Conversely, antagonism occurs when the activity of the combination is inferior to that of the most active component alone, implying that one agent interferes with or diminishes the antibacterial effect of the other [9].

The main adverse effects associated with intravenous administration of fosfomycin include hypokalemia and hypernatremia (due to the high sodium content of intravenous fosfomycin), predominantly mild to moderate in severity and only infrequently lead to treatment discontinuation [7, 8]. In the multicenter observational FORTRESS study, hypokalemia and hypernatremia were reported in 26.4% and 15.2%, respectively; however, these events were predominantly mild to moderate in severity. Overall, fosfomycin was discontinued in 12 patients [8].

Growing clinical evidence suggests that combination regimens including fosfomycin, particularly when associated with agents such as cefiderocol or sulbactam, may improve clinical and microbiological outcomes in patients with CRAB infections. Although *A. baumannii* is intrinsically resistant to fosfomycin, mainly because of reduced membrane permeability (e.g., absence or alteration of glycerophosphate transporters), the presence of fosfomycin-inactivating enzymes (such as FosA-like enzymes), and mutations involving target enzymes such as MurA, fosfomycin may still exert synergistic activity when used in combination therapies [1, 7].

The apparent discrepancy between the limited intrinsic in vitro activity of fosfomycin against *A. baumannii* and the favorable outcomes reported in some clinical studies may be explained by potential indirect effects. These include pharmacodynamic synergy with partner antibiotics, enhanced intracellular penetration in infected tissues, and possible immunomodulatory properties [10]. Experimental data suggest that fosfomycin may modulate host inflammatory responses by reducing pro-inflammatory cytokine production and influencing leukocyte function, which could be particularly relevant in severe CRAB infections where host-mediated damage contributes substantially to disease severity. However, this hypothesis remains speculative in the context of CRAB and requires further investigation [11, 12].

Currently, there is a lack of guidance regarding the use of fosfomycin in clinical practice, particularly for patients with CRAB infections, for which therapeutic options remain extremely limited.

Therefore, we conducted a narrative review to evaluate the role of fosfomycin in combination therapy for CRAB infections, with the aim of summarizing current evidence and clarifying whether fosfomycin may represent a viable therapeutic option in this setting.

## Methods

A literature review was conducted using the PubMed database, employing the search terms “fosfomycin” AND “Acinetobacter” without restrictions on publication date or language.

Eligible studies included in vitro investigations, animal models, clinical observational studies, clinical trials, and relevant case reports evaluating fosfomycin in combination with other antimicrobial agents against CRAB. References of the included articles were screened to identify additional relevant publications.

The findings were synthesized and presented as a narrative review, focusing on the efficacy, synergistic potential, and clinical outcomes associated with fosfomycin-containing regimens in the treatment of CRAB infections.

As this review was designed as a narrative rather than a systematic review, no formal risk-of-bias assessment was performed.

## Results

A total of 217 articles were initially identified and screened. After applying the inclusion criteria, 48 studies were included in the final analysis. Of these, 27 reported in vitro data on fosfomycin-based combination therapies, mainly involving synergy testing (*n* = 23) [13–39], while 22 provided in vivo (*n* = 2) [40, 41] and clinical data (*n* = 20) [32, 42–60]. One case presented both in vitro and clinical data [32].

### In vitro data

In vitro data on fosfomycin combinations with other antimicrobial agents against *A. baumannii* isolates are summarized in Table 1. Overall, in vitro evidence suggests that fosfomycin-based combination regimens may exert synergistic or additive activity against *A. baumannii* isolates, including CRAB, although results remain heterogeneous and strongly influenced by the antimicrobial partner and testing methodology. Across the available studies, the most consistent synergistic interactions were observed with sulbactam, imipenem, and, to a lesser extent, colistin [14, 18–25, 28, 32, 34]. In particular, fosfomycin–sulbactam combinations demonstrated high rates of synergy in checkerboard assays, frequently confirmed by time-kill experiments and supported by a very limited number of pharmacokinetic/pharmacodynamic (PK/PD) models and hollow-fiber infection studies, which showed substantial reductions in bacterial burden and enhanced bacterial killing compared with monotherapy [14, 24, 28, 34]. Mohd Sazlly Lim et al. demonstrated through Monte Carlo simulations that fosfomycin enhanced the bactericidal activity of sulbactam. In combination regimens including sulbactam 3 g every 8 h plus fosfomycin 4 g, 6 g, or 8 g every 8 h, the predicted bacterial killing ranged from approximately 1 to 3.5 log10 CFU/mL. Furthermore, in a hollow-fibre infection model, the combination of fosfomycin with continuous-infusion sulbactam resulted in an approximately 2–4 log10 reduction in bacterial burden within the first 24 h [23].

**Table 1:** In vitro results of fosfomycin combinations against *Acinetobacter baumannii*

Authors, Year of Publication, and Country	Test method	Number of CRAB; period of isolation; range MIC (mg/L) for FOS*	Resistance and synergy association results
Martinez-Martinez et al. 1996, Spain [13]	Checkerboard assay	34; 1990–1995; *≥*128 (microdilution)	AMK 31/34 R (NCCLS) S: 15/34 An: 0/34 TOB 33/34 R (NCCLS) S: 11/34 An: 0/34 IMI 7/34 R (NCCLS) S: 1/34 An: 0/34
Santimaleeworagun et al. 2011, Thailand [14]	Checkerboard assay, Time-kill assay	8; 2008; 128–512 (agar dilution)	CST 0/8 R (CA-SFM) Checkerboard assay S: 1/8 Ad: 2/8 I: 5/8 An: 0/8 SUL 6/8 R (CA-SFM) Checkerboard assay S: 6/8 Ad: 0/8 I: 2/8 An: 0/8 Time-kill assay S: 6/8
Zhang et al. 2013, China [15]	Checkerboard assay	25; na; > 512 (broth microdilution)	Polymyxin B (na) S: 4/25 Ad: 11/25 I: 10/25 An: 0/25 MIN (na) S: 3/25 Ad: 14/25 I: 8/25 An: 0/25
Leite et al. 2016, Brazil [16]	Checkerboard assay, 2Well, Time-kill assay	20; 2002–2012; 32 to 128 (broth microdilution)	GEN 11/20 R and AMK 1/20 R (CLSI) Checkboard assay S: 0/20 Ad: 1/20 I: 19/20 An: 0/20 2Well S: 13/20 (GEN) and 19/20 (AMK) I: 7/20 (GEN) and 2/20 (AMK) An: 0/20 Time-kill assay S: 13/20 (GEN) and 19/20 (AMK)
Menegucci et al. 2016, Brazil [17]	Checkerboard assay and Monte Carlo simulation for PK/PD	6; 2008–2012; 64 to 512 (agar dilution)	MEM (EUCAST) S: 1/6 Ad: 5/6 I: 1/6 An: 0/6 Polymyxin B 3/6 R (EUCAST) S: 1/6 Ad: 4/6 I: 1/6 An: 0/6 MEM + Polymyxin S: 5/6 Ad: 1/6 I: 0/6 An: 0/6
Wei et al. 2016, China [18]	Checkerboard assay	50; 2013; 8 to *≥* 32; (agar dilution)	CST 25/50 R (CLSI) S: 25/50 Ad/I: 22/50 An: 3/50
Zhu et al. 2018, China [19]	Checkerboard assay	21; 2014; 32 to 256 (broth microdilution)	IMI 21/21 R (CLSI) S: 12/21 Ad: 3/21 I: 6/21 An: 0/21 CST 3/21 R (CLSI) S: 0/21 Ad: 2/21 I: 19/21 An: 0/21
Singkham-in et al. 2018, Thailand [20]	Checkerboard assay and Time-kill assay	23; 2010–2011 and 2015–2016; 128 to 256 (broth microdilution)	IMI 23/23 R (CLSI) Checkboard assay S: 15/23 Ad: 7/23 I: 1/23 An: 0/23 Time-kill assay S: 8/9 MEM 23/23 R (CLSI) Checkboard assay S: 0/23 Ad: 3/23 I: 20/23 Ant: 0/23
Leelasupasri et al., 2019, Thailand [21]	Checkerboard assay	15; 2014–2015;64 to 246 (E-test)	CST 0/15 R (CLSI) S: 4/15 Ad: 7/15 I: 4/15 An: 0/15
Bian et al. 2019, China [22]	Checkerboard assay	9; 2014; na	CST 0/9 R (CLSI) S: 1/9 Ad: 0/9 I: 0/9 An: 0/9
Flamm et al. 2019, U.S. [23]	Checkboard assay and Time-kill assay	5; 2015; na	Checkboard assay AMK (na) S: 2/5 Ad: 3/5 I: 0/5 An: 0/5 MEM (na) S: 1/5 Ad: 3/5 I: 0/5 An: 0/5 CST (na) S: 2/5 Ad: 2/5 I: 0/5 An: 0/5 TIG (na) and MIN (na) S: 0/5 Ad: 4/5 I: 0/5 An: 0/5 MEM Time-kill assay S: 1/1
Mohd Sazlly Lim et al. 2021, Australia [24]	Checkboard assay, Time-kill assay, Monte Carlo simulations	50; 2011–2013; 128 to 2,048 (agar dilution)	SUL R (na) Checkboard assay S: 37/50 Ad: 12/50 I: 1/50 An: 0/50 Time-kill assay S: 2/2
Singkham-in et al. 2022, Thailand [25]	Checkboard assay and Time-kill assay	2; 2018; 128 and 256 (agar dilution)	IMI 2/2 R (CLSI) Checkboard assay S: 2/2 Ad: 0/2 I: 0/2 An: 0/2 Time-kill assay S: 1/2 MEM 2/2 R (CLSI) Checkboard assay S: 0/2 Ad: 2/2 I: 0/2 An: 0/2
Xu et al. 2022, China [26]	Checkerboard assay	25; 2011–2019; 512 to 1024 (agar dilution)	IMI/REL 3/3 R S: 3/3 Ad: 0/3 I: 0/3 An: 0/3
Boncompagni et al. 2022, Italy [27]	Checkerboard assay and quantitative antibiofilm assays	5; na; 64 to 1024 (agar dilution)	CST (na) Checkboard assay S: 0/5 Ad: 3/5 I: 2/5 An: 0/5 Quantitative antibiofilm assays S: 3/3
Mohd Sazlly Lim et al. 2022, Australia [28]	Hollow-fibre infection model	3; 2011–2013; 128 to 256 (agar dilution)	SUL (na) S 3/3
Adaleti et al. 2022, Turkey [29]	Checkboard assay	6; 2021–2022; *≥* 128 (agar dilution)	MEM R 6/6 (EUCAST) S: 3/6 Ad: 2/6 I: 1/6 An: 0/6
Palombo et al. 2023, Italy [30]	E-test synergy	2; 2021–2022; 32 to ≥ 256 (test strips)	FDC 1/2 R (EUCAST) S: 1/2 Ad: 1/2 I: 0/2 An: 0/2 SUL R (na) S: 0/2 Ad: 0/2 I: 2/2 An: 0/2
Quraini et al. 2023, India [31]	Checkboard assay and Time-kill assay	8; 2019–2020; 32 to ≥ 128	MEM R 8/8 (EUCAST) Checkboard assay S: 7/8 Ad: 1/8 I: 0/8 An: 0/8 Time-kill assay S: 1/1 AMK R 7/8 (EUCAST) Checkboard assay S: 0/8 Ad: 0/8 I: 8/8 An: 0/8 Time-kill assay S: 1/1 TIG R 7/8 (EUCAST) Checkboard assay S: 0/8 Ad: 7/8 I: 0/8 An: 0/8 Time-kill assay S: 1/1
Müderris et al. 2024, Turkey [32]	Checkboard assay and E-test synergy	21; na; 32 to 512 (agar dilution)	MEM R 21/21 (EUCAST) Checkboard assay S: 11/21 Ad: 8/21 I: 2/21 An: 0/21 E-test S: 0/21 Ad: 0/21 I: 7/21 An: 14/21 CST R (na) Checkboard assay S: 6/21 Ad: 7/21 I: 7/21 An: 1/21 E-test S: 2/21 Ad: 0/21 I: 3/21 An: 16/21
Prenika et al. 2025, South Africa [33]	E-test synergy	30; 2023; 8 *≤* to 512 (E-test)	CST R (na) S: 0/30 Ad: 15/30 I: 7.5/30 An: 8/30 TIG R 1/30 (FDA) S: 7.5/30 Ad: 7.5/30 I: 15/30 An: 0/30 MEM R 30/30 (CLSI) S: 0/30 Ad: 0/30 I: 26/30 An: 4/30
Jitmuang et al. 2025, Thailand [34]	Checkboard assay and Time-kill assay	62; 2016–2020; 64 to > 512 (broth microdilution)	SUL R (na) Checkboard assay S: 26/62 Ad: 25/62 I: 11/62 An: 0/62 Time-kill assay S: 2/3 (*n* = 3 of 26 with synergy effect at checkboard assay)
Zocche et al. 2026, Italy [35]	E-test synergy	13; 2019–2022; 32 to > 256 (broth microdilution)	SUD R 4/13 (CLSI) S: 0/13 Ad: 3/13 I: 10/13 An: 0/13

Synergistic interactions were interpreted according to the fractional inhibitory concentration index (FICI) as follows: synergy (S), FICI < 0.5; additive effect (Ad), FICI between 0.5 and 1; indifference (I), FICI > 1 to 4; and antagonism (An), FICI > 4

*AMK* amikacin, *CA-SFM* Comité de l’Antibiogramme de la Société Française de Microbiologie, *CRAB* carbapenem resistant *A. baumannii*, *CST* colistin, *FDC* cefiderocol, *FOS* fosfomycin, *IMI/REL* imipenem/relebactam, *MIN* minocycline, *na* not available, *NCCLS* National Committee for Clinical Laboratory Standards, *R* Resistance, *SUD* sulbactam/durlobactam, *SUL* sulbactam, *TIG*: tigecycline, *TOB* tobramycin

* MIC values should be interpreted with caution when determined using methods other than the reference agar dilution technique, particularly the glucose-6-phosphate (G6P)-supplemented agar dilution method, which is considered the gold standard for fosfomycin susceptibility testing. Alternative methodologies, including gradient diffusion and automated systems, may yield discrepant results and can affect the accuracy and comparability of susceptibility data across studies. This limitation applies to the majority of studies included in the present review

Similarly, combinations with imipenem demonstrated reproducible synergistic activity across several studies, whereas fosfomycin combined with meropenem generally resulted in additive or indifferent effects, with synergistic interactions reported less frequently [13, 17, 19, 20, 23, 25, 26, 29, 31–33].

Fosfomycin–colistin combinations yielded variable results, ranging from synergistic to indifferent interactions, although some studies demonstrated enhanced activity against biofilm-associated isolates and suppression of polymyxin-resistant subpopulations [14, 18, 21–23, 27, 32, 33]. Also, Fedrigo et al. observed that the addition of fosfomycin in combination with polymyxin B plus meropenem significantly restricted the selective amplification of polymyxin B-resistant subpopulation [61].

In addition, Boncompagni et al. demonstrated that the combination of fosfomycin and colistin, at concentrations achievable through nebulized administration, exhibited significant synergistic activity against biofilm-forming MDR Gram-negative pathogens. Notably, inhaled fosfomycin was estimated to achieve epithelial lining fluid concentrations exceeding 1200 mg/L, supporting the potential role of aerosolized fosfomycin-based regimens in difficult-to-treat respiratory infections [27].

Synergy with aminoglycosides, particularly amikacin, was also reported in selected studies [13, 16, 23], while combinations with tigecycline or minocycline mainly produced additive effects [15, 23, 31, 33]. Fosfomycin combined with amikacin showed synergistic activity in approximately 25% of tested isolates (17/67). Inhaled amikacin and fosfomycin have been investigated as a potential combination therapy for the treatment of MDR Gram-negative pneumonia, including CRAB, particularly in ventilator-associated pneumonia (VAP). In vitro studies have demonstrated that the amikacin–fosfomycin combination can reduce MIC values compared with either agent alone, with evidence of variable but often synergistic interactions, supporting further evaluation of this aerosolized dual-antibiotic strategy in critically ill patients [62].

Data regarding fosfomycin combined with cefiderocol remain extremely limited. To date, only one small in vitro study has specifically evaluated this combination against two CRAB isolates, demonstrating synergistic and additive interactions in one isolate each. Notably, synergistic activity was observed in the cefiderocol-resistant strain [30].

**Fig. 1:**
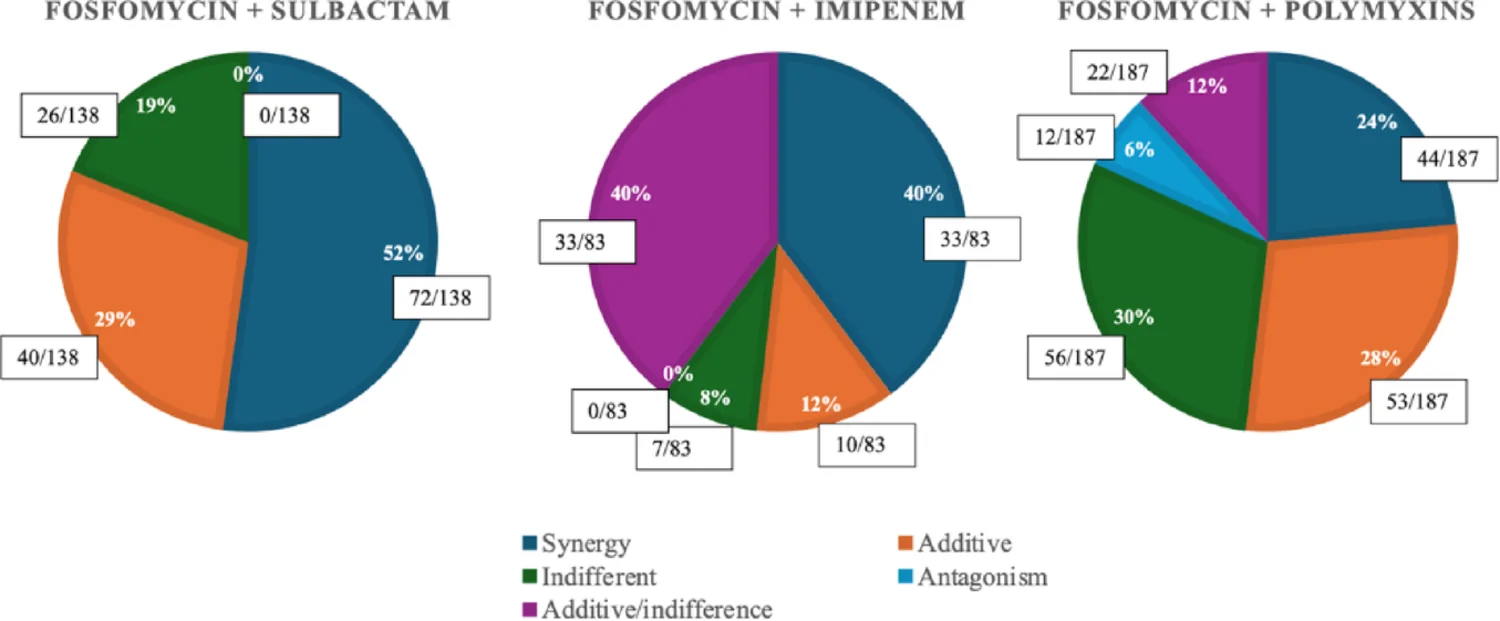
In vitro activity of fosfomycin-based combination regimens against carbapenem-resistant *Acinetobacter baumannii* (CRAB). The values were derived from the synergy testing data reported in Table 1. In vitro data demonstrated the highest rates of synergistic activity for fosfomycin-containing combinations with sulbactam, followed by imipenem. Fosfomycin–sulbactam combinations showed synergy in 52.2% of isolates (72/138) and additive effects in 29.0%, whereas fosfomycin–imipenem combinations demonstrated synergy in 40.0% of isolates (33/83). Combinations with polymyxins (colistin/polymyxin B) exhibited lower synergistic activity (24%; 44/187) and substantial heterogeneity across studies, although additive effects were observed in 28.0–40.0% of isolates

In summary, the highest overall synergy rates were observed with sulbactam-containing regimens, with synergistic effects reported in approximately 52.2% of isolates (72/138), while an additional 29.0% showed additive activity, (see Fig. 1). Fosfomycin combined with imipenem demonstrated synergy in about 40.0% of isolates (33/83). For polymyxins (colistin/polymyxin B), pooled results showed synergy in approximately 24.0% of isolates (44/187) and additive effects in 28%, although heterogeneity among studies was substantial. Time-kill assay was performed in a limited number of cases, usually confirming the synergistic effect of the checkerboard assay in most of the tested combinations (55/68, 79.4%) [14, 16, 20, 23–25, 31, 34].

Notably, substantial discrepancies were observed between different in vitro methods, especially between checkerboard and E-test assays, highlighting the lack of methodological standardization in synergy testing [32]. Importantly, no consistent antagonism was reported across most studies. Beyond direct antibacterial activity, mechanistic investigations suggested that fosfomycin-containing combinations may interfere with cell wall recycling pathways, increase membrane permeability, and potentiate partner antibiotics through complementary mechanisms of action [25].

Fosfomycin has also been investigated in combination with bacteriophage therapy against *A. baumannii*, including biofilm-associated infections, although overall efficacy remained limited [36, 37]. Within biofilm models, the bacteriophage–imipenem combination demonstrated the highest antibacterial activity [36]. In addition, fosfomycin was also evaluated against CRAB in combination with novel agents. Saini et al. demonstrated that efflux pump inhibition by the small molecule IITR08367 significantly enhances the antibacterial efficacy of fosfomycin by increasing intracellular drug accumulation [38]. Zhu et al. showed that polymyxin B-functionalized fosfomycin liposomes improve targeting toward Gram-negative bacteria and produce a strong synergistic antibacterial effect [39].

However, these aggregated findings should be interpreted with caution due to marked heterogeneity in study designs, susceptibility profiles, synergy-testing methodologies, and interpretive criteria across studies.

### In vivo animal models

 In vivo murine-thigh infection model showed more efficacy of the colistin and fosfomycin combination than colistin monotherapy, although synergistic inhibition was only found in 1/12 strains [40]. In a mouse model of CRAB pneumonia, the combination of fosfomycin and colistin significantly reduced bacterial load in lung tissue compared with monotherapy, demonstrating bactericidal and synergistic effects [41].

### Clinical studies

Table 2 summarizes the available clinical evidence regarding fosfomycin-containing combination regimens for the treatment of CRAB infections. Overall, the available evidence is limited and heterogeneous, comprising predominantly retrospective observational studies, case series, and case reports, with only three clinical trials specifically evaluating fosfomycin-containing regimens [42–44].

**Table 2:** Clinical study results including fosfomycin combination for the treatment of carbapenem-resistant *A. baumannii*

Authors, Year of Publication, and Country	Type of Study, years	Infections and FOS combinations	Outcomes
*Trials*			
Sirijatuphat et al. 2014 [42]	Open-label randomized controlled	Pneumonia (37 vs. 35), BSI (2 vs. 3), UTI (3 vs. 2), SSTI (2 vs. 1), IAI (2 vs. 4), CNS (0 vs. 1), other (1 vs. 1): 47 COL + FOS (4 g q12h) vs. 47 COL	A fosfomycin-containing regimen was associated with a more favorable microbiological response (100% vs. 84.5%, *p* = 0.023), non-significant clinical outcomes (62.8% vs. 56.4%, *p* = 0.578), and a non-significant lower mortality (16.3% vs. 23.1%, *p* = 0.578).
Hakamifard et al. 2023, Iran [43]	Single-center open-label randomized controlled trial, 2019 to 2020	55 VAP: 28 pts (MEM iv + CST iv + Inh CST) vs. 27 pts (MEM iv + CST iv + Inh TOB + Inh FOS [80 mg q12h])	The mean treatment duration in the TOB + FOS group was lower (*P* = 0.001) Higher reduction after 72 h in Clinical Pulmonary Infection Score in the TOB + FOS group (*p* = 0.005) Microbiological eradication at day 7 was higher in the TOB + FOS group (92.6% vs. 32%, *p* < 0.001)
Hakamifard et al. 2025, Iran [44]	Triple-blind randomized clinical trial	VAP: 37 pts (MEM iv + CST iv + Inh CST) vs. 39 pts (MEM iv + CST iv + Inh CST + Inh FOS [80 mg q12h])	Differences in treatment failure rates between the groups were not statistically significant (18.9% vs. 7.7%, *p* = 0.19) CST/FOS group showed higher microbiological eradication (48.7% vs. 35.1%) Treatment duration shorter in CST/FOS group (8.97 days vs. 14.06 days, *p* < 0.001)
*Observational studies*			
Russo et al. 2020, Italy [45]	Observational, prospective, multicenter study; 2017 to 2020	44/180 pneumonia: 11 CST + FOS 8 carbapenem + TIG + FOS 7 CST + TIG + FOS 7 RIF + FOS 6 TIG + FOS 3 carbapenem + FOS 2 aminoglycoside + FOS	Fosfomycin-containing regimen (HR 0.04, CI 95% 0.01–0.13, *p* < 0.001) was associated with 30-day survival.
Casarotta et al. 2022, Italy [46]	Observational, retrospective Monocentric study, 2020 to 2021	8/32 VAP: FOS + AMP/SUL + TIG+ iv CST + inh CST	100% microbiological eradication
Thampithak et al. 2022, Thailand [47]	Observational, retrospective Monocentric study, 2019 to 2020	52 Pneumonia, 3 IAI, 2 UTI, 4 BSI, 11 SSTI: FOS ± (6 carbapenem/64 CST/4 LFX/3 aminoglycoside/11 AMP-SUL/1 TIG/1 TMP/SMX)	The 14-day all-cause mortality rate was 54.8%; only in 10 cases (14.3%) was one active antibiotic
Mazzitelli et al. 2023, Italy [48]	Observational, retrospective, monocentric study, 2021 to 2022	53 BSI and 58 pneumonia: 11/111 treated with FOS regimen 8 FDC + FOS 3 CST + FOS	FOS did not influence the outcomes
Russo et al. 2023, Italy [49]	Observational, retrospective single-center study, 2020 to 2022	73 pts with COVID VAP with CRAB BSI 20/73 treated with FOS regimen: 3 CST + FOS 3 CST + MEM + FOS 6 FDC + FOS 3 FDC + FOS + TIG 3 FDC + FOS + TIG + MEM 1 FDC + FOS + AMP/SUL 1 FDC + FOS + TIG + MEM + TMP/SMX	FDC-containing regimens (HR 0.34, 95% CI 0.18–0.56; *P* < 0.001) and FDC + FOS therapy (HR 0.22, 95% CI 0.1– 0.55; *P* < 0.001) were independently associated with 30-day survival
Russo et al. 2024, Italy [50]	Observational, retrospective multicenter study, 2017 to 2022	60 VAP, 22 BSI, 16 Catheter-related infection, 4 other infections: FOS ± (8 MEM/48 CST/18 TIG/18 AMP/SUL/2 TMP/SMX/54 FDC/22 Inh CST)	30-day mortality 47% FDC-containing regimen was associated with survival (63% vs. 42%, *P* = 0.032) while the CST-containing regimen with clinical failure (63% vs. 33%, *P* = 0.003)
Oliva et al. 2024, Italy [51]	Observational, retrospective Monocentric study, 2019 to 2023	Catheter-related infection and pneumonia: 7 FDC + FOS 1 FDC + AMP/SUL + FOS 1 FDC + TIG + FOS 1 CST + AMP/SUL + FOS 1 CST + FOS	No significant difference in survival analysis (11 FOS vs. 33 no FOS) The overall analysis, including CRAB and Kp, FOS group showed significantly higher clinical cure, early improvement and microbiological eradication than no FOS group Mortality did not differ according to the different FOS partners
Russo et al. 2026, Italy [52]	Observational, prospective, multicenter study; 2023 to 2024	BSI: 54 FOS + FDC	FDC plus FOS was significantly associated with clinical success (*p* = 0.024) FDC–FOS combination therapy did not significantly differ from FDC monotherapy in terms of 14-/30-day mortality or clinical failure
*Case series and case report*			
Bavaro et al. 2021, Italy [53]	Case series, 2020 to 2021	4/8 CRAB (2 catheter-related infections, 1 VAP, 1 BSI): 2 FDC + FOS+TIG 1 FDC + FOS 1 FDC + CST + FOS	The authors evaluated FDC in switch therapy combined with FOS in 4/8 cases. Recovery in 3 cases, 1 case microbiological eradication with death
Marino et al. 2022, Italy [54]	Case series, 2022	1 BSI, FDC + FOS	Clinical cure
Assimakopoulos et al. 2023, Greece [55]	Case series, 2020 to 2022	8/20 BSI treated with FOS regimen: 1 CST + TMP/SMX + FOS 1 CST + FOS 1 AMK + AMP/SUL + CST + FOS 2 CST + TIG + FOS 1 TGC + AMP/SUL + AMK + FOS 1 CST + MEM + GEN + TMP/SMX + FOS 1 CST + TIG + AMK + TMP/SMX + FOS	Inclusion of FOS in the combination therapeutic regimen was associated with significantly better survival as compared to non-FOS-containing regimens (*p* = 0.0051).
Müderris et al. 2024, Turkey [32]	Retrospective case series, unspecified	BSI: 1 FOS + CST + MEM HAP: 1 FOS + CST + MEM + VAN	1 Microbiological eradication 1 Treatment failure
Mellon et al. 2012, France [56]	Case report	CNS AMP/SUL + RIF + FOS	Full recovery
Kerz et al. 2018, Germany [57]	Case report	CNS: MEM + CST + FOS	Death for a non-infectious condition
Aykota et al. 2020, [58]	Case report	1 (BSI, UTI and SSTI): IMI + TIG + FOS	Full recovery
Pallotto et al. 2025, Italy [59]	Case report	Prosthetic joint infection: prosthesis removal and CIP + MIN + FOS	Clinical and microbiological cure
Lin et al. 2025, China [60]	Case report	VAP: Inh phage therapy + iv polymyxin B, iv amikacin, and iv fosfomycin	Clinical improvements in 8 days

*AMK* amikacin, *AMP* ampicillin, *BSI* bloodstream infection, *CIP* ciprofloxacin, *CNS* central nervous system, *CRAB* carbapenem resistant *A. baumannii,* *CST* colistin, *FDC* cefiderocol, *FOS* fosfomycin, *GEN* gentamicin, *HAP* hospital-acquired pneumonia. IAI intrabdominal infection, *Inh* inhaled, *iv* intravenous, *LFX* levofloxacin, pts: patients, *RIF* rifampin, *SSTI* skin and soft tissue infection, *SU*D sulbactam/durlobactam, *SUL* sulbactam, *TIG* tigecycline, *TMP/SMX* TOB tobramycin, *TMP/SMX* trimethoprim/sulfamethoxazole, *UTI* urinary tract infection

Most studies involved critically ill patients with severe CRAB infections, particularly hospital-acquired pneumonia (HAP), including VAP, BSIs, and catheter-related infections.

Furthermore, fosfomycin-based combination regimens have also been evaluated in infections caused by pan-drug-resistant (PDR) isolates, with some studies reporting improved clinical outcomes [46, 47, 55].

In particular, Assimakopoulos et al. observed significantly improved survival among patients receiving fosfomycin-containing combination therapy for PDR CRAB bacteremia (*p* = 0.0051). The comparison groups received different multidrug regimens, including combinations of colistin, tigecycline, carbapenem, aminoglycosides, and trimethoprim-sulfamethoxazole, whereas fosfomycin was additionally included in the therapeutic regimen of eight patients [55]. However, the available evidence remains extremely limited, and the specific contribution of fosfomycin is difficult to ascertain, given that it was administered in combination with multiple different agents in most cases.

Three clinical trials evaluated fosfomycin-containing regimens in CRAB infections [42–44]. In the randomized, open-label trial conducted by Sirijatuphat et al., 99 adult patients with carbapenem-resistant *Acinetobacter baumannii* infections were enrolled, of whom 94 were included in the final analysis (47 per treatment arm). The study was performed in a single tertiary-care hospital and included a critically ill population with severe healthcare-associated infections, predominantly ventilator-associated pneumonia, alongside a smaller proportion of bloodstream and other nosocomial infections. Most patients were managed in intensive care units, and a high proportion required mechanical ventilation, reflecting substantial disease severity and comorbidity burden. Patients were randomized to receive either colistin monotherapy (5 mg colistin base activity/kg/day, adjusted for renal function) or colistin plus fosfomycin (4 g every 12 h). The addition of fosfomycin was associated with significantly higher microbiological eradication rates, while no significant differences were observed in clinical outcomes or 28-day mortality between the two treatments [42].

Two subsequent trials evaluated adjunctive inhaled fosfomycin-based therapies in CRAB VAP [43, 44]. In both studies, fosfomycin-containing regimens resulted in improved microbiological eradication and shorter treatment duration. Fosfomycin combination therapy was not associated with reductions in treatment failure or mortality [43, 44].

However, the largest amount of evidence derives from observational multicenter studies conducted in Italy.

The largest body of evidence derives from four Italian multicentre observational studies conducted by Russo et al., which collectively included 730 patients with severe infections due to CRAB, of whom 220 received combination therapy with fosfomycin [45, 49, 50, 52]. In these studies, fosfomycin-containing regimens, particularly combinations including cefiderocol, were frequently associated with improved clinical outcomes and survival [45, 49, 50, 52]. In the prospective multicenter study by Russo et al. involving 157 patients with CRAB pneumonia, fosfomycin-containing combinations were was associated with 30-day survival (HR 0.04, 95% CI 0.01–0.13; *p* < 0.001) [45].

In a retrospective multicenter cohort evaluating 102 patients with severe CRAB infections treated with combination therapy including intravenous fosfomycin, cefiderocol-containing regimens were associated with higher survival rates, whereas colistin-containing combinations were associated with clinical failure. However, since all enrolled patients received fosfomycin-containing regimens, the observed differences in outcomes were more likely attributable to the companion agent, particularly cefiderocol, rather than to fosfomycin itself [50].

In another retrospective study including 73 patients with COVID-19-associated VAP and CRAB bacteremia treated with various multi-antibiotic colistin- or cefiderocol-based regimens, with or without fosfomycin, the cefiderocol–fosfomycin combination was independently associated with improved 30-day survival [49]. However, the presence of COVID-19 may have acted as a potential confounding factor, possibly influencing the robustness and generalizability of the observed results.

More recently, in the Italian multicenter prospective ITACA study, Russo et al. analyzed 398 patients with CRAB BSIs, including 54 treated with fosfomycin-based combinations. Fosfomycin-containing combinations, particularly cefiderocol plus fosfomycin, were associated with higher clinical success rates in patients with CRAB BSIs after propensity score matching [52]. Notably, clinical success was defined not only by survival but also by early infection control, including fever resolution, ≥ 50% reduction in procalcitonin after 3 days of therapy, and absence of persistent CRAB-related clinical signs. However, in analyses restricted to cefiderocol-treated patients, cefiderocol–fosfomycin combination therapy did not differ significantly from cefiderocol monotherapy in 14-/30-day mortality or clinical failure, suggesting that the observed benefit may primarily reflect improved early clinical response rather than a clear survival advantage [52].

In another Italian study, Oliva et al. reported no significant survival differences between fosfomycin-containing and non-fosfomycin-containing regimens in CRAB infections alone, although the overall cohort, including both CRAB and *Klebsiella pneumoniae* infections, showed higher rates of clinical cure and microbiological eradication in the fosfomycin group [51]. Likewise, Mazzitelli et al. found no significant impact of fosfomycin on outcomes in patients receiving cefiderocol- or colistin-based therapy [48].

## Discussion

The treatment of infections caused by CRAB remains one of the greatest therapeutic challenges in contemporary clinical microbiology and infectious diseases. Available antimicrobial options are severely limited, and in many cases no fully active agent is identified in vitro, leading clinicians to rely on combination regimens as salvage therapy in critically ill patients, including those infected with PDR strains.

Among potential adjunctive agents, fosfomycin has attracted increasing interest despite the intrinsic resistance of *A. baumannii*, which is primarily related to reduced membrane permeability, alterations or absence of glycerophosphate transport systems, enzymatic inactivation, and target-site modifications [1, 7]. Nevertheless, the present review demonstrates that both experimental and clinical evidence support a possible role for fosfomycin when administered in combination with other anti-CRAB agents.

 In vitro studies indicate that fosfomycin may exert additive or synergistic activity, particularly when combined with sulbactam, imipenem, aminoglycosides, and colistin, although results remain highly heterogeneous [18–20, 24, 32, 34]. Among the evaluated regimens, fosfomycin–sulbactam combinations consistently demonstrated the most robust synergistic activity across checkerboard assays, time-kill analyses, and pharmacodynamic models. By contrast, interactions with tigecycline and minocycline were more frequently additive or indifferent. Importantly, substantial methodological variability across studies, especially between checkerboard and gradient diffusion techniques, limits direct comparability and underscores the need for standardized approaches to synergy testing. Despite this heterogeneity, no consistent antagonism has been reported, supporting the microbiological compatibility of fosfomycin within combination regimens. Although limited in number, in vivo animal studies also support a potential benefit of fosfomycin-based combinations.

Clinical evidence remains largely observational and heterogeneous, comprising predominantly retrospective cohorts and case series, with only a limited number of clinical trials. Overall, fosfomycin has most commonly been administered in combination with cefiderocol, colistin, and sulbactam-containing regimens in critically ill patients with severe CRAB infections, including VAP and BSIs.

Available randomized clinical trials suggest that adjunctive intravenous or inhaled fosfomycin does not significantly reduce mortality or clinical failure, although it consistently improves both the rate and speed of microbiological eradication, particularly in respiratory infections [42–44]. These findings may reflect the fact that enrolled patients were critically ill and frequently died from factors extending beyond the infection itself, potentially masking any survival benefit attributable to antimicrobial therapy.

Some observational studies, particularly multicenter Italian cohorts, suggest an association between fosfomycin-containing regimens and improved clinical outcomes, including survival in CRAB pneumonia and bacteremia. However, these findings are inconsistent across studies, and other analyses have not confirmed a clear benefit. The interpretation of these data is further complicated by the frequent co-administration of multiple active agents, which precludes attribution of outcomes to fosfomycin alone. For example, Casarotta et al. reported microbiological eradication in eight patients with CRAB VAP treated with a combination protocol including high-dose ampicillin/sulbactam, high-dose tigecycline, intravenous and inhaled colistin, and fosfomycin. However, given the use of multiple concomitant active agents and the absence of an appropriate comparator specifically evaluating fosfomycin, the independent contribution of fosfomycin to these outcomes cannot be determined [46].

Moreover, the apparent benefit associated with fosfomycin-containing regimens may partly reflect indication bias, center-specific expertise, greater access to newer agents such as cefiderocol, and the use of intensified combination strategies in selected patients, rather than a direct independent effect of fosfomycin itself.

Among the evaluated combinations, fosfomycin plus cefiderocol appears to be one of the most promising strategies, supported by observational data suggesting improved early clinical response and, in some studies, a survival benefit [49, 50, 52]. Nevertheless, microbiological evidence supporting this combination remains extremely limited, with only preliminary in vitro data demonstrating occasional synergy or additivity, including against cefiderocol-resistant isolates [30]. Although observational findings are encouraging, the current evidence remains insufficient to establish whether fosfomycin provides a true additive clinical benefit when combined with cefiderocol or simply represents a marker of more aggressive combination-based therapeutic approaches.

### Summary of evidence

The available evidence on fosfomycin-containing regimens for the treatment of CRAB is limited and highly heterogeneous, comprising predominantly in vitro studies, experimental animal models, and observational clinical data, with only a small number of clinical trials.

In vitro data suggest that fosfomycin may exert synergistic or additive effects when combined with several anti-CRAB agents, most consistently with sulbactam, imipenem, and, to a lesser extent, colistin. However, results are markedly variable across studies and strongly influenced by testing methodology and antimicrobial partner. No consistent antagonism has been reported, although methodological heterogeneity limits comparability and reproducibility.

Clinical evidence remains largely observational and non-randomized, with a substantial risk of confounding due to the frequent use of multiple concomitant active agents. Fosfomycin has most commonly been used in combination with cefiderocol, colistin, carbapenems, and sulbactam-containing regimens in critically ill patients with severe CRAB infections. While several retrospective and multicenter studies, particularly from Italian cohorts, suggest an association between fosfomycin-containing regimens and improved clinical outcomes and survival, other studies have not confirmed these findings.

Current evidence suggests that the combination of fosfomycin with cefiderocol may represent one of the most promising therapeutic strategies against CRAB, with observational studies indicating improved early clinical response and a potential survival benefit. However, both microbiological and clinical evidence supporting this regimen remain limited, precluding definitive conclusions regarding its superiority over cefiderocol monotherapy.

Overall, fosfomycin should be considered an adjunctive agent within combination therapy for CRAB infections, with its clinical role remaining uncertain. The most promising signals appear to involve combinations with cefiderocol and sulbactam, but definitive conclusions are precluded by the low quality and heterogeneity of the available evidence.

### Limitations

This review has several limitations. First, the available evidence is highly heterogeneous and largely derives from non-randomized studies, posing a substantial risk of confounding. Most clinical investigations were retrospective, single-center studies with limited sample sizes, thereby reducing the overall strength and generalizability of the findings. In addition, the frequent use of multidrug combination regimens precludes robust attribution of clinical outcomes specifically to fosfomycin.

Clinical evidence is further limited by the scarcity of randomized controlled trials and the predominance of observational data involving critically ill patients treated with multiple concomitant anti-CRAB agents. Consequently, the independent contribution of fosfomycin within combination regimens remains difficult to define.

Considerable heterogeneity also affects the preclinical literature. In vitro studies differ substantially with respect to synergy testing methodologies, interpretive criteria, and experimental conditions, making direct comparison across studies challenging. Similarly, in vivo evidence remains limited, with few comparative animal studies and a lack of standardized experimental models.

Publication bias cannot be excluded, particularly regarding case reports and small case series, which are inherently more likely to describe favorable outcomes and may therefore overestimate the perceived benefit of fosfomycin-containing regimens. Finally, another important limitation is the narrative design of the review itself, which may have introduced selection bias in study inclusion and interpretation. Moreover, the absence of standardized definitions for synergy and clinical response across studies further limits the comparability of the available evidence.

## Conclusion

Although fosfomycin lacks reliable activity as monotherapy against carbapenem-resistant *Acinetobacter baumannii*, accumulating in vitro, in vivo, and clinical evidence suggests a potential adjunctive role within combination regimens. Experimental studies frequently demonstrate synergistic or additive interactions, particularly with sulbactam, carbapenems, and colistin, although substantial methodological heterogeneity limits cross-study comparability.

Clinical evidence remains largely observational and is confounded by the concomitant administration of multiple active agents. Nevertheless, several multicenter studies have suggested an association between fosfomycin-containing combinations and improved early clinical response and, in selected cohorts, improved survival. Among the available strategies, the combination with cefiderocol appears particularly promising, although current supporting evidence remains limited and insufficient to support definitive conclusions.

Overall, fosfomycin may be considered a potential adjunct in carefully selected combination regimens for severe CRAB infections. Well-designed prospective studies and adequately powered randomized clinical trials are urgently needed to clarify its true clinical impact and define its optimal therapeutic role.

## Data Availability

No datasets were generated or analysed during the current study.
